# Lipopolysaccharide uptake is augmented in lipopolysaccharide‐tolerant mouse macrophage‐like cells via increased CD14 expression

**DOI:** 10.1002/2211-5463.70261

**Published:** 2026-04-26

**Authors:** Saeka Nishihara, Takayuki Manabe, Mika Jouta, Kiyoshi Kawasaki

**Affiliations:** ^1^ Laboratory of Biochemistry, Graduate School of Drug Discovery Sciences Osaka Metropolitan University Japan; ^2^ Faculty of Pharmaceutical Sciences Doshisha Women's University Kyoto Japan; ^3^ Department of Nursing Asahikawa Medical University Asahikawa Hokkaido Japan

**Keywords:** glycosylphosphatidylinositol‐anchored CD14, innate immunity, LPS hyporesponsiveness

## Abstract

Lipopolysaccharide (LPS) tolerance can be recognized as a modulation of innate immune responses rather than merely a hyporesponsiveness to LPS, with CD14 being crucial for both LPS uptake and LPS signaling. In this study, we observed that LPS‐tolerant mouse macrophage‐like cells, in which LPS‐induced TNF‐α and IFN‐β production was suppressed, exhibited a dramatic increase in surface CD14 expression. Also, we found that LPS uptake was enhanced in LPS‐tolerant mouse macrophage‐like cells, but not when treated with an anti‐CD14 antibody. While previous studies have reported increased CD14 expression and enhanced LPS uptake in LPS‐tolerant cells, our findings reveal that overexpressed CD14 in LPS‐tolerant mouse macrophage‐like cells is responsible for the enhanced LPS uptake in these cells.

## Graphical abstract

In normal (nontolerant) cells, CD14 is crucial for both LPS uptake and LPS signaling. In LPS‐tolerant cells, in which LPS‐induced TNF‐α and IFN‐β production is suppressed, there is a dramatic increase in surface CD14 expression. The overexpressed CD14 in LPS‐tolerant cells is responsible for the enhanced LPS uptake without inducing pro‐inflammatory signaling in these cells.

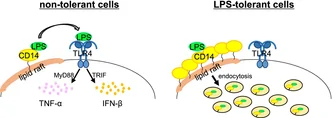

**Abbreviations**
DRM: detergent resistant membrane
ELISA: enzyme‐linked immunosorbent assay
Geo MFI: geometric mean of fluorescence intensity
GPI: glycosylphosphatidylinositol
HRP: horseradish peroxidase
LPS: lipopolysaccharide
PE: phycoerythrin
qRT‐PCR: quantitative reverse transcription‐polymerase chain reaction
SDS: sodium dodecyl sulfate
TBP: TATA‐box binding protein

In the initial phase of microbial infection, innate immune responses, such as the production of inflammatory cytokines by macrophages, are activated to eliminate the invading pathogens [1]. CD14, a molecule attached to the plasma membrane through a glycosylphosphatidylinositol (GPI) anchor, plays a crucial role in recognizing lipopolysaccharide (LPS) [2]. As CD14 is a GPI‐anchored membrane protein, it transduces LPS‐induced cellular signals by interacting with other signaling molecules, including the TLR4‐MD‐2 complex. Following the transfer of LPS from the LPS‐binding protein to CD14, CD14 transfers the LPS to TLR4‐MD‐2, which dimerizes and initiates cellular signaling, including two major pathways: the MyD88‐ and TRIF‐dependent signaling pathways [2, 3, 4, 5]. CD14 is required for the endocytosis of LPS that enables subsequent TRIF‐dependent signaling and is also required for the MyD88‐dependent signaling pathway [6, 7, 8, 9]. When these LPS‐induced innate immune responses become excessive, they can lead to septic shock syndrome, including extensive tissue damage and serious systemic disorders with a high mortality rate [10]. Therefore, pathophysiological adaptations to regulate excessive inflammatory responses are crucial mechanisms for host defense against the deleterious consequences of microbial infection. One of the classic examples of such a protective mechanism is LPS tolerance, a phenomenon in which immune cells exposed to low concentrations of LPS for prolonged periods enter a transient hyporesponsive state and exhibit reduced responses to subsequent LPS stimulation [11]. However, LPS‐tolerant cells retain some abilities to combat infection, such as bacterial phagocytosis and reactive oxygen species production [12, 13]. Therefore, LPS tolerance can now be recognized as a form of cellular function modulation rather than simply as hyporesponsiveness.

CD14 plays a crucial role in the establishment of tolerance to LPS [14], but its functional role in LPS‐tolerant cells has not been well characterized. It has been previously reported that LPS‐tolerant macrophages retain the ability for LPS uptake [15], but the relationship between CD14 expression and LPS uptake remains unclear. On the contrary, the surface expression of CD14 in LPS‐tolerant cells varies depending on the cell type and experimental conditions, with reports of decreased [16], increased [17], or unchanged levels [18]. In this study, we investigated the functional role of CD14 in LPS‐tolerant mouse macrophage‐like RAW264.7 cells.

## Materials and methods

### Reagents

LPS from *Escherichia coli* O111:B4 was purchased from List Biological Laboratories (Campbell, CA, USA). LPS from *Escherichia coli* O55:B5 conjugated to Alexa Fluor 488 (LPS‐Alexa488), anti‐mouse TNF‐α antibody (clone XT3), and biotinylated anti‐mouse TNF‐α antibody (clone XT22) were purchased from Thermo Fisher Scientific (Waltham, MA, USA). Recombinant mouse TNF‐α was purchased from TONBO Biosciences (San Diego, CA, USA). Anti‐mouse IFN‐β antibody, biotinylated anti‐mouse IFN‐β antibody (clone MIB‐5E9.1), and recombinant mouse IFN‐β were purchased from BioLegend (San Diego, CA, USA). Anti‐mouse CD14 antibody (clones rmC5‐3 and 4C1) and goat anti‐rat Ig conjugated to phycoerythrin (PE) were purchased from BD Biosciences (Franklin Lakes, NJ, USA). Anti‐mouse TLR4 antibody (clone 25), anti‐rat IgG antibody conjugated to horseradish peroxidase (HRP), anti‐mouse IgG antibody conjugated to HRP, normal mouse IgG, and normal rat IgG_1_ were purchased from Santa Cruz Biotechnology (Dallas, TX, USA). Anti‐Histone H3 antibody (clone 96C10) was purchased from Cell Signaling Technology (Danvers, MA, USA). Chlorpromazine was purchased from Sigma‐Aldrich (St. Louis, MO, USA). Anti‐mouse CD36 antibody (clone CRF D‐2712) was purchased from Hycult Biotech (Uden, Netherlands).

### Cell culture

The mouse macrophage‐like cell line RAW264.7 was purchased from DS Pharma Biomedical (Osaka, Japan) and maintained at low passage numbers prior to use in experiments. Identity and purity of the cell line were periodically authenticated, including within the last 3 years, based on morphological inspection and verification of characteristic LPS‐induced responses, such as pro‐inflammatory cytokine secretion. Cells were cultivated in high‐glucose Dulbecco's modified Eagle's medium (Sigma‐Aldrich, MO, USA) supplemented with 10% (vol/vol) heat‐inactivated (56 °C for 30 min) fetal bovine serum (Sigma‐Aldrich), 100 units·mL^−1^ penicillin (Thermo Fisher Scientific, MA, USA), and 100 μg·mL^−1^ streptomycin (Thermo Fisher Scientific) at 37 °C in a humidified environment with 5% CO_2_, as previously described [19].

### Analysis of cytokine production

RAW264.7 cells were pretreated with or without 10 ng·mL^−1^ LPS for 12 h. After washing the cells thrice, fresh culture medium was added, and the cells were incubated for 2 h. Next, the cells were treated with the indicated concentrations of LPS for 6 h. Supernatants were then collected, and TNF‐α and IFN‐β concentrations were measured by sandwich enzyme‐linked immunosorbent assay (ELISA) using the following method.

Individual wells of an Immulon 2HB flat‐bottom 96‐well microtiter plate (Thermo Fisher Scientific) were coated with 100 μL of 5.0 μg·mL^−1^ anti‐mouse TNF‐α antibody or 0.5 μg·mL^−1^ anti‐mouse IFN‐β antibody at 4 °C overnight. A biotinylated anti‐mouse TNF‐α antibody (0.25 μg·mL^−1^) or biotinylated anti‐mouse IFN‐β (1.0 μg·mL^−1^) antibody was mixed with an equal volume of the supernatant. Recombinant mouse TNF‐α and IFN‐β were used as standards. The biotinylated antibody was detected using an avidin‐biotin system with the VECTASTAIN Elite ABC Kit (Vector Laboratories, Newark, CA, USA) and TMB Peroxidase Substrate Kit (Vector Laboratories). The absorbance at 450 nm was measured after stopping the color reaction with peroxidase by adding 100 μL of 1 N sulfuric acid.

### Analysis of CD14 gene expression

CD14 gene expression was analyzed by quantitative reverse transcription‐polymerase chain reaction (qRT‐PCR). RAW264.7 cells were treated with or without 10 ng·mL^−1^ LPS for 12 h, after which 1 × 10^6^ cells were collected, and total RNA was purified using a High Pure RNA Isolation Kit (Roche Diagnostics, Rotkreuz, Switzerland). The total RNA (200 ng) was reverse transcribed to cDNA using a PrimeScript RT reagent Kit (Takara Bio Inc., Shiga, Japan). The cDNA (1 μL) was used as a template for PCR using LuminoCt SYBR Green qPCR Ready Mix (Sigma‐Aldrich) and an Eco Real‐time PCR System (Illumina, San Diego, CA, USA). Specific primers for CD14 (forward, 5′‐GGCTTGTTGCTGTTGCTTC‐3′; reverse, 5′‐CAGGGCTCCGAATAGAATCC‐3′) and TATA‐box binding protein (TBP) (forward, 5′‐CAGTTACAGGTGGCAGCATGA‐3′; reverse, 5′‐TAGTGCTGCAGGGTGATTTCAG‐3′) were used. Gene expression was quantified using the standard curve method based on quantification cycle values.

### Analysis of the cell surface expression of CD14

RAW264.7 cells were treated with or without 10 ng·mL^−1^ LPS for 12 h. After treatment, the cells were collected and resuspended to achieve a concentration of 2 × 10^7^ cells·mL^−1^. To prevent nonspecific staining, the cell suspension (100 μL) was incubated with 5 μg of normal mouse IgG at 4 °C for 10 min. After incubation, 2 μL of anti‐mouse CD14 antibody (clone rmC5‐3) or normal rat IgG_1_ (for isotype control) was added, and the mixture was incubated at 4 °C for 60 min in the dark. After washing the cells by centrifugation, 1 μL of goat anti‐rat Ig conjugated to PE (secondary antibody) was added, and the mixture was incubated at 4 °C for 30 min in the dark. Subsequently, the expression of CD14 on the cell surface was analyzed using a FACSCalibur flow cytometer (BD Biosciences), and the geometric mean of fluorescence intensity (Geo MFI) of 10 000 cells was determined using the CellQuest Pro software (BD Biosciences).

### Analysis of CD14 and TLR4 expression

RAW264.7 cells were treated with 10 ng·mL^−1^ LPS for the indicated times. After treatment, the cells were collected and resuspended in phosphate‐buffered saline (137 mm NaCl, 2.7 mm KCl, 1.47 mm KH_2_PO_4_, 8.1 mm Na_2_HPO_4_) containing 0.1% sodium dodecyl sulfate (SDS) and a protease inhibitor cocktail (Roche Diagnostics). The cell suspension was sonicated several times and used as the whole‐cell lysates. Alternatively, the detergent resistant membrane (DRM) fraction was prepared as previously described [20], with slight modifications. Briefly, the collected cells were resuspended in 800 μL of 25 mm MES (pH 6.5) containing 150 mm NaCl, 1% Triton X‐100, 2 mm NaF, 2 mm Na_3_VO_4_ and a protease inhibitor cocktail and mixed by inversion at 4 °C for 1 h. Following centrifugation, the pellet was resuspended in 150 μL of 10 mm Tris/HCl (pH 7.5) containing 150 mm NaCl, 1.85% octyl‐glucoside, 1 mm NaF, 1 mm Na_3_VO_4_ and a protease inhibitor cocktail and sonicated for 10 s and incubated at 4 °C for 30 min. After centrifugation, the supernatant was collected as the DRM fractions.

Equal amounts of proteins from whole‐cell lysates or DRM fractions were separated by SDS/polyacrylamide gel electrophoresis and transferred onto a nitrocellulose membrane. The expression of CD14, TLR4, and Histone H3 was detected by western blotting using anti‐mouse CD14 antibody (clone rmC5‐3, diluted 1:10000), anti‐mouse TLR4 antibody (diluted 1:1000), and anti‐Histone H3 antibody (diluted 1:1000). Blots were developed using a SuperSignal West Femto Maximum Sensitivity Substrate (Thermo Fisher Scientific), and signals were visualized using a LAS‐3000 mini (FujiFilm, Tokyo, Japan) or Amersham Imager 600 (GE Healthcare Life Sciences, Chicago, IL, USA). Densitometric analysis was performed using the ImageJ software (National Institutes of Health, Bethesda, MD, USA) and normalized to Histone H3 as an internal control.

### Analysis of cellular uptake of LPS

RAW264.7 cells were treated with or without 10 ng·mL^−1^ LPS for 12 h. After washing the cells thrice, fresh culture medium was added, and the cells were incubated for 2 h. Next, the cells were treated with 1 μg·mL^−1^ LPS‐Alexa488. For chlorpromazine or antibody treatment, the cells were pretreated with the indicated concentrations of chlorpromazine or antibody for 30 min before treatment with LPS‐Alexa488. After pretreatment, the culture medium was replaced with that containing LPS‐Alexa488 along with the same concentrations of chlorpromazine or antibody. Following treatment with LPS‐Alexa488 for 2 h, the cells were analyzed using a Nikon A1R confocal laser scanning microscope (Nikon, Tokyo, Japan) as described previously [19] or a FACSCalibur flow cytometer. In the flow cytometry analysis, the Geo MFI of 10 000 cells was determined using the CellQuest Pro software.

### Statistical analysis

Statistical significance was determined using Student's *t*‐test, with *P* < 0.05 considered statistically significant. When the *F*‐test indicated unequal variances, Welch's *t*‐test was used instead.

## Results

### LPS‐tolerant RAW264.7 cells exhibit increased cell surface CD14 expression compared with nontolerant cells

We examined the production of TNF‐α and IFN‐β, major products of the MyD88‐ and TRIF‐dependent pathways, respectively, by RAW264.7 cells pretreated with or without 10 ng·mL^−1^ LPS for 12 h, followed by stimulation with different concentrations of LPS (1, 10, 100, or 1000 ng·mL^−1^). When the cells were stimulated with LPS without pretreatment, the production of TNF‐α and IFN‐β were increased in a dose‐dependent manner (Fig. 1A,B). In contrast, the production of TNF‐α and IFN‐β by cells pretreated with LPS was significantly reduced to 16.8% and 51.8%, respectively, in response to subsequent stimulation with 1000 ng·mL^−1^ LPS, compared with that in cells without pretreatment (Fig. 1A,B). These results indicate that the pretreatment successfully induced LPS tolerance in RAW264.7 cells. Therefore, we defined RAW264.7 cells pretreated with 10 ng·mL^−1^ LPS for 12 h as LPS‐tolerant cells and those without LPS pretreatment as nontolerant cells.

**Fig. 1:**
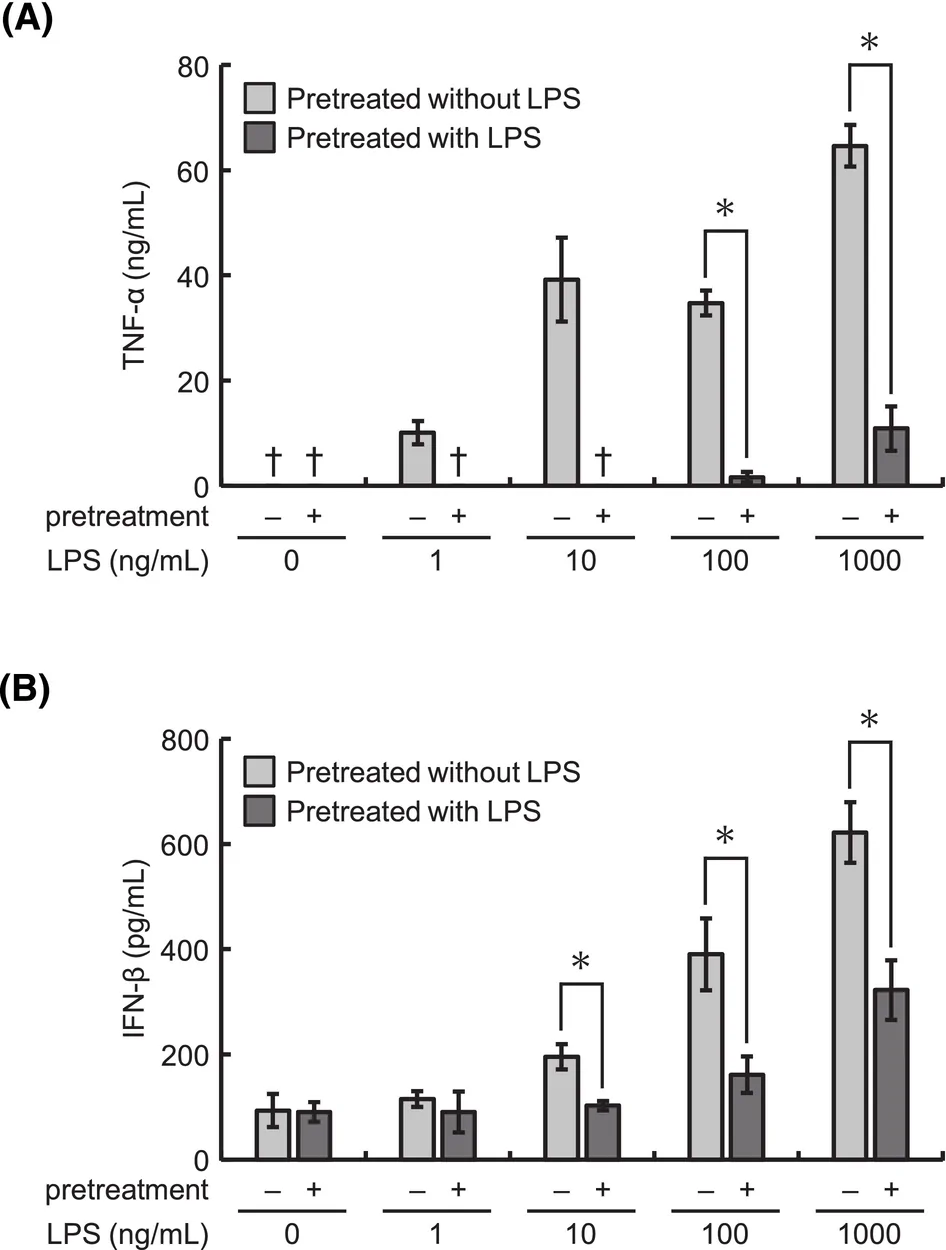
Lipopolysaccharide (LPS)‐induced cytokine production in RAW264.7 cells is decreased by pretreatment with LPS. RAW264.7 cells were pretreated with (+) or without (−) 10 ng mL^−1^ LPS for 12 h, followed by stimulation with the indicated concentrations (ng mL^−1^) of LPS. Concentrations of TNF‐α (A) and IFN‐β (B) in the culture supernatants, measured by ELISA, are shown as means ± standard deviations from triplicate analyses. Daggers (†) indicate that TNF‐α was not detected (less than 0.625 ng mL^−1^). Statistical comparison of cytokine production levels between cells pretreated with or without LPS was performed using Student's *t*‐test (*P < 0.05). (A) The *P*‐values for these comparisons were as follows: *P* = 5.4 × 10^−5^ and 1.9 × 10^−4^ for following LPS stimulation with 100 and 1000 ng mL^−1^, respectively. (B) The *P*‐values for these comparisons were as follows: *P* = 0.0071, 0.013, and 0.0064 for following LPS stimulation with 10 100, and 1000 ng mL^−1^, respectively.

We examined CD14 expression in LPS‐tolerant RAW264.7 cells. CD14 gene expression was examined by qRT‐PCR, revealing an approximately 13.6‐fold increase in LPS‐tolerant cells compared with that in nontolerant cells (Fig. 2A). CD14 expression on the cell surface was also examined by flow cytometry. The Geo MFI of LPS‐tolerant cells stained for cell surface CD14 was 5.6‐fold higher than that of nontolerant cells, indicating that CD14 expression on the cell surface was increased in LPS‐tolerant cells (Fig. 2B). Furthermore, CD14 expression in whole‐cell lysates prepared from LPS‐treated cells was examined by western blotting. LPS treatment increased CD14 levels, with the highest levels observed 6–12 h after treatment (Fig. 2C). In contrast, TLR4 levels were not altered by LPS treatment for up to 24 h (Fig. 2C). As GPI‐anchored CD14 is constitutively present in lipid rafts, where it facilitates LPS signaling in a steady‐state condition [21], we examined CD14 levels in DRM fractions, which are commonly used as models of lipid rafts [22], prepared from LPS‐treated cells. CD14 levels in DRM fractions increased following LPS treatment, with the highest levels observed 12 h after treatment (Fig. 2D). Successful DRM preparation was confirmed by the presence of the DRM marker flotillin‐1 and the absence of the non‐DRM marker transferrin receptor in the DRM fractions (Fig. S1). Therefore, CD14 expression increased in response to LPS treatment, and its translocation to the lipid rafts was maximal at 12 h after treatment. Taken together, CD14 expression was dramatically increased in LPS‐tolerant RAW264.7 cells, and the increased CD14 was also localized within lipid rafts on the cell surface, which are appropriate sites for GPI‐anchored CD14.

**Fig. 2:**
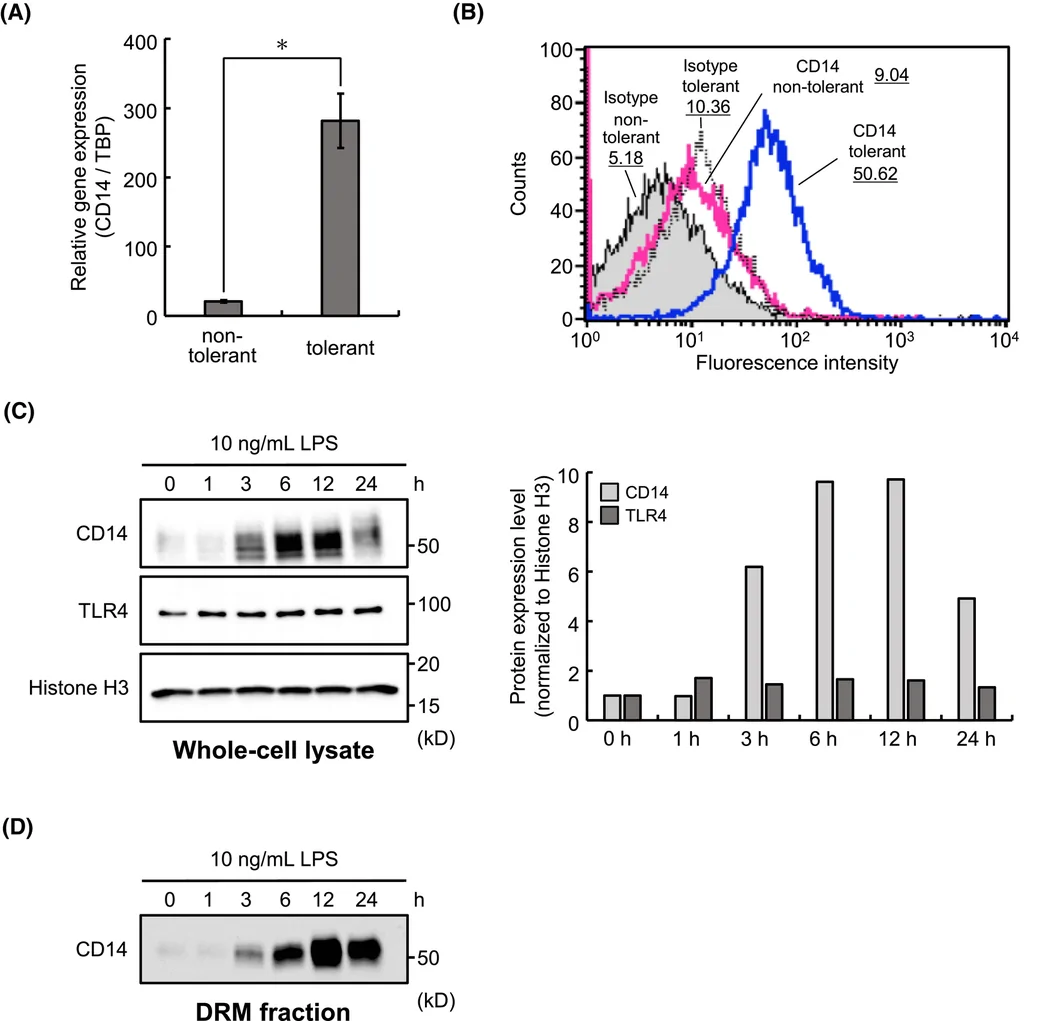
CD14 expression is increased on the surface of lipopolysaccharide (LPS)‐tolerant RAW264.7 cells. (A and B) LPS tolerance was induced in RAW264.7 cells by pretreatment with 10 ng mL^−1^ LPS for 12 h. (A) CD14 gene expression in LPS‐tolerant or nontolerant cells, measured by qRT‐PCR and normalized to the housekeeping gene TBP, is shown as means ± standard deviations from triplicate analyses. Statistical comparison was performed using Welch's *t*‐test (*P* = 0.011; *P < 0.05). (B) CD14 expression on the surface of LPS‐tolerant or nontolerant cells was analyzed by staining with anti‐CD14 antibody (normal rat IgG_1_ was used as an isotype control) and PE‐conjugated secondary antibody. The fluorescence intensities of the cells analyzed by flow cytometry are shown. The underlined values in the histogram indicate Geo MFIs. (C and D) RAW264.7 cells were treated with 10 ng mL^−1^ LPS for the indicated times. CD14, TLR4, and Histone H3 in whole‐cell lysates (C) and CD14 in the DRM fractions (D) were analyzed by western blotting. Densitometric analysis was performed, normalized to Histone H3, and values are expressed relative to the 0 h time point in (C).

### LPS endocytosis is enhanced in LPS‐tolerant RAW264.7 cells compared with that in nontolerant cells

As CD14 is crucial for the endocytosis of LPS, we examined the uptake of fluorescence‐labeled LPS in LPS‐tolerant RAW264.7 cells. As shown in Fig. 3A, enhanced cellular uptake of LPS was observed in LPS‐tolerant cells compared with that in nontolerant cells. A punctate LPS pattern was detected mainly in the cytoplasm, suggesting that LPS was taken up by active transport mechanisms, such as endocytosis. Quantification of the fluorescence intensity of LPS showed that the Geo MFI of LPS‐tolerant cells was 2.71‐fold higher than that of nontolerant cells (Fig. 3B). We also examined whether the enhanced uptake of LPS in LPS‐tolerant RAW264.7 cells was mediated by endocytosis. It has been previously reported that LPS uptake by RAW264.7 cells is mediated by clathrin‐dependent endocytosis [23]. Thus, we examined the uptake of LPS in LPS‐tolerant RAW264.7 cells treated with chlorpromazine, which inhibits clathrin‐dependent endocytosis [24]. The uptake of LPS by LPS‐tolerant cells was reduced by chlorpromazine treatment compared with cells without treatment (Fig. 3C). These results indicate that LPS‐tolerant RAW264.7 cells exhibit enhanced LPS uptake compared with that in nontolerant cells, probably via clathrin‐dependent endocytosis.

**Fig. 3:**
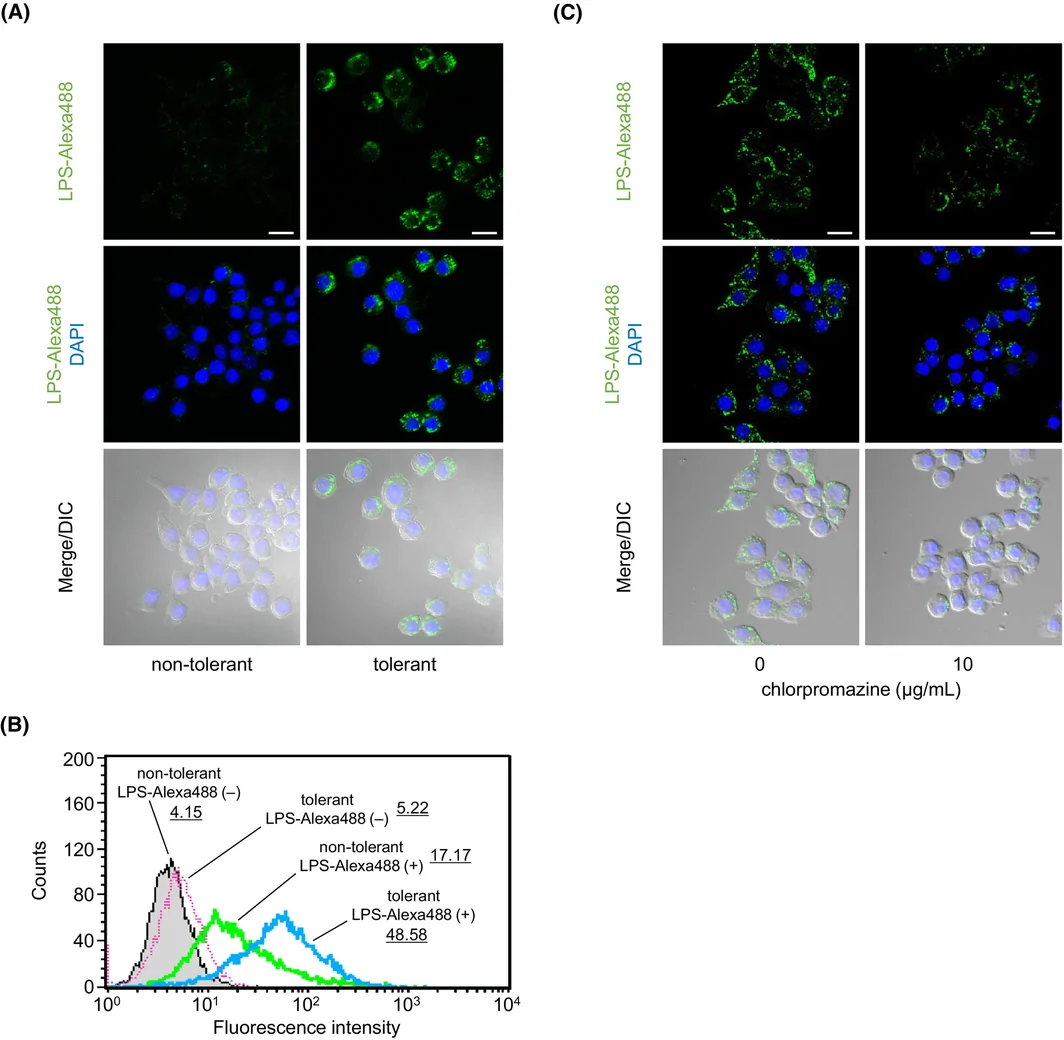
Lipopolysaccharide (LPS)‐tolerant RAW264.7 cells exhibit enhanced LPS endocytosis. LPS tolerance was induced in RAW264.7 cells by pretreatment with 10 ng mL^−1^ LPS for 12 h. LPS‐tolerant or nontolerant cells were treated with (+) or without (−) LPS‐Alexa488. (C) LPS‐tolerant cells were pretreated with 10 μg mL^−1^ chlorpromazine before LPS‐Alexa488 treatment. (A and C) The cells were analyzed by confocal microscopy. LPS‐Alexa488 (green), nuclei stained with DAPI (blue), and merged images with DIC are shown. Scale bars (20 μm) are shown in the top panel; all panels are at the same magnification. (B) The fluorescence intensities of the cells analyzed by flow cytometry are shown. The underlined values in the histogram indicate Geo MFIs. DAPI, 4′,6‐diamidino‐2‐phenylindole; DIC, differential interference contrast.

### Enhanced LPS uptake in LPS‐tolerant RAW264.7 cells is mediated by increased CD14 expression

To determine whether the enhanced LPS uptake in LPS‐tolerant RAW264.7 cells is mediated by the increased CD14 expression, we examined the effects of anti‐CD14 antibody (clone 4C1), which blocks LPS binding to CD14 [25], on LPS uptake in LPS‐tolerant cells. As shown in Fig. 4A, internalized LPS fluorescence was evident in LPS‐tolerant cells without antibody treatment but not in cells treated with 2.5 μg·mL^−1^ anti‐CD14 antibody. Furthermore, internalized LPS fluorescence was quantified by flow cytometry. The amount of internalized LPS in LPS‐tolerant cells was decreased in a dose‐dependent manner following treatment with an anti‐CD14 antibody (Fig. 4B). Similarly, the amount of internalized LPS in nontolerant cells decreased following treatment with anti‐CD14 antibody (Fig. 4B). The Geo MFI of LPS‐tolerant and nontolerant cells treated with 5.0 μg·mL^−1^ anti‐CD14 antibody was reduced to 42.3 and 51.2%, respectively, compared with that in cells without antibody treatment (Fig. 4B). These observations are consistent with the consideration that the increased CD14 expression in LPS‐tolerant cells functions similarly in LPS uptake to CD14 in nontolerant cells. To assess the specificity of CD14 involvement in LPS uptake by LPS‐tolerant cells, we also examined the potential contribution of CD36, a scavenger receptor known to mediate the recognition, uptake, and clearance of pathogens as well as their cell wall components, including LPS [26, 27]. Internalized LPS fluorescence in LPS‐tolerant cells treated with 10 μg·mL^−1^ anti‐CD36 antibody, which has been previously confirmed to inhibit LPS uptake by macrophages [28], was comparable to that in cells without antibody treatment (Fig. 4C). Taken together, these results indicate that the enhanced uptake of LPS in LPS‐tolerant RAW264.7 cells is mediated by increased CD14 expression in these cells.

**Fig. 4:**
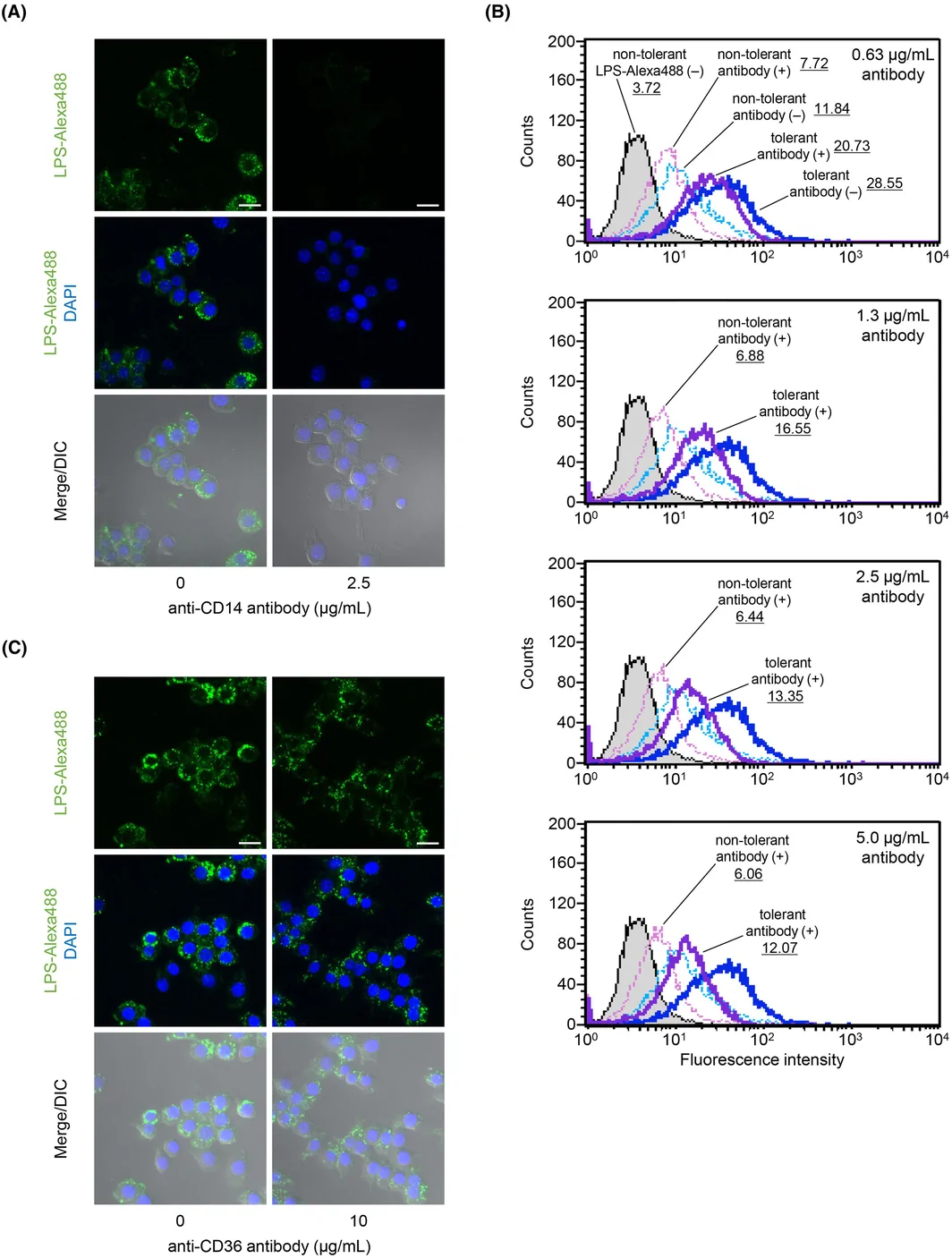
Enhanced lipopolysaccharide (LPS) uptake in LPS‐tolerant RAW264.7 cells is inhibited by treatment with anti‐CD14 antibody. LPS tolerance was induced in RAW264.7 cells by pretreatment with 10 ng mL^−1^ LPS for 12 h. (A and C) LPS‐tolerant cells were treated with LPS‐Alexa488 in the presence or absence of (A) 2.5 μg mL^−1^ anti‐CD14 antibody (clone 4C1) and (C) 10 μg mL^−1^ anti‐CD36 antibody. The cells were analyzed using confocal microscopy. LPS‐Alexa488 (green), nuclei stained with DAPI (blue), and merged images with DIC are shown. Scale bars (20 μm) are shown in the top panel; all panels are at the same magnification. (B) LPS‐tolerant or nontolerant cells were treated with LPS‐Alexa488 in the presence of the indicated concentrations of anti‐CD14 antibody (clone 4C1). The fluorescence intensities of the cells analyzed by flow cytometry are shown. For clarity of comparison, the results are separated into four panels for each concentration of anti‐CD14 antibody: 0.63, 1.3, 2.5, and 5.0 μg mL^−1^ from top to bottom. The same histograms are presented in all panels for direct comparison: nontolerant cells treated with or without LPS‐Alexa488 and LPS‐tolerant cells treated with LPS‐Alexa488. The underlined values in the histograms indicate Geo MFIs. DAPI, 4′,6‐diamidino‐2‐phenylindole; DIC, differential interference contrast.

## Discussion

In this study, we found that LPS‐tolerant RAW264.7 cells, in which LPS‐induced TNF‐α and IFN‐β production was suppressed, exhibited a dramatic increase in surface CD14 expression. To our knowledge, this is the first demonstration of such a pronounced increase in CD14 expression in LPS‐tolerant cells. Furthermore, we observed that LPS endocytosis was enhanced in LPS‐tolerant RAW264.7 cells. Several studies have reported increased CD14 expression and enhanced LPS uptake in LPS‐tolerant cells [15, 17], but their interrelationship has not been directly investigated. We revealed that overexpressed CD14 in LPS‐tolerant RAW264.7 cells is responsible for the enhanced LPS uptake in these cells. RAW264.7 cells are a well‐established macrophage model widely used for analyzing innate immune responses [29]. In the present study, RAW264.7 cells were employed to reduce cellular heterogeneity, thereby enabling analysis under a controlled cellular context. Moreover, the TLR4–CD14 axis is highly conserved across species, supporting the broader relevance of our findings. However, as these results are currently limited to RAW264.7 cells, further investigation in other murine or human macrophage cell lines or primary cells is warranted to confirm the generality of the observed phenomenon.

It has been previously reported that CD14‐mediated LPS uptake is insufficient to trigger LPS signaling [30]. In our study, the increased expression of CD14 in LPS‐tolerant RAW264.7 cells likely contributed to enhanced LPS uptake independent of LPS signaling. In LPS‐tolerant cells, MyD88‐ or TRIF‐dependent signaling pathways are known to be suppressed by negative feedback mechanisms, including the induction of microRNAs, such as miR155, miR146, and miR9 [11, 31], possibly uncoupling LPS uptake from pro‐inflammatory signaling. LPS uptake is an important step for its removal and detoxification [9]. Therefore, overexpressed CD14 in LPS‐tolerant cells may play a distinct role as a facilitator of LPS clearance in modulating the function of these cells, contributing to host defense against microbial infection without triggering inflammatory signaling. Our findings revealed a potential function of regulating CD14 expression in LPS‐tolerant macrophages.

## Conflict of interest

The authors declare no conflict of interest related to this study.

## Author contributions

T.M. and K.K. conceived and designed the project. S.N. and M.J. acquired the data. S.N., T.M., and M.J. analyzed and interpreted the data. S.N. and K.K. wrote the paper.

## Supporting information


**Fig. 1** Expression of flotillin‐1 and transferrin receptor in DRM fractions.

## Data Availability

The data that support the findings of this study are available in all figures and/or the supplementary material of this article.
