# Supplementary material for: Lipopolysaccharide uptake is augmented in lipopolysaccharide‐tolerant mouse macrophage‐like cells via increased CD14 expression
Source: FEBS Open Bio. 2026 Apr 26:10.1002/2211-5463.70261. Online ahead of print. doi: 10.1002/2211-5463.70261 (PMC13398632; doi:10.1002/2211-5463.70261)
Supplement: Supplementary file 1 — Fig. 1 Expression of flotillin‐1 and transferrin receptor in DRM fractions. [file FEB4-9999-0-s001.pdf]

# Supplementary Figure 1

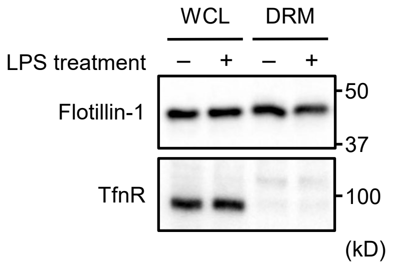


**Supplementary Figure 1. Expression of flotillin-1 and transferrin receptor in DRM fractions.** RAW264.7 cells were treated with (+) or without (−) 10 ng/mL LPS for 6 h. Flotillin-1 and transferrin receptor (TfnR) in whole-cell lysates (WCL) and DRM fractions (DRM) were analyzed by western blotting using anti-flotillin-1 antibody (1:1,000, Cell Signaling Technology, Cat# 18634) and anti-TfnR antibody (1:1,000, Cell Signaling Technology, Cat# 46222).
